# Clinical and molecular features of innate and acquired resistance to anti-PD-1/PD-L1 therapy in lung cancer

**DOI:** 10.18632/oncotarget.23315

**Published:** 2017-12-15

**Authors:** Shalin Shah, Kevin Wood, Brian Labadie, Brian Won, Ryan Brisson, Theodore Karrison, Thomas Hensing, Mark Kozloff, Riyue Bao, Jyoti D. Patel, Jason J. Luke

**Affiliations:** ^1^ Department of Medicine, NorthShore University HealthSystems, Chicago, IL, USA; ^2^ Department of Medicine, University of Chicago, Chicago, IL, USA; ^3^ Department of Public Health Sciences, University of Chicago, Chicago IL, USA; ^4^ Center for Research Informatics and Department of Pediatrics, University of Chicago, Chicago, IL, USA

**Keywords:** non-small cell lung cancer, anti-PD-1/PD-L1 therapy, acquired resistance, immunotherapy, innate resistance

## Abstract

**Hypothesis:**

The majority of non-small cell lung cancer (NSCLC) patients treated with anti-PD-1/PD-L1 therapy develop either innate or acquired resistance. Across tumor types, the “T cell-inflamed” tumor microenvironment correlates with clinical response to immunotherapy. We hypothesize that clinical characteristics may be predictive of resistance and that “T cell-inflamed” NSCLC tumors can be identified by gene expression profiling.

**Results:**

Of 93 patients, 36 (38.7%) had innate resistance and 57 (61.3%) had initial benefit to immunotherapy. Innate resistance was associated with non-smokers (*p* = 0.013), more involved disease sites (*p* = 0.011), more prior therapy (*p* = 0.001), and a lower albumin level (*p* = 0.014). Among patients with initial benefit, factors associated with subsequent progression-free survival included higher Karnofsky Performance Status (KPS) (*p* = 0.004) and lower depth of response to anti-PD-1 therapy (*p* = 0.003). A “T cell-inflamed” microenvironment was identified in 42% of TCGA adenocarcinoma samples versus 21.0% of squamous cell.

**Discussion:**

Specific clinical characteristics appear to be predictive of either innate or acquired resistance to anti-PD-1/PD-L1 therapy. A “T cell-inflamed” tumor was more common in adenocarcinoma than squamous histology.

**Methods:**

A retrospective review of NSCLC patients treated with anti-PD-1/PD-L1 monotherapy. Patients with innate resistance to anti-PD-1/PD-L1 therapy (defined as progression at first CT evaluation) were compared to patients with initial clinical benefit. Among those with initial clinical benefit, we identified prognostic factors for time to progression (acquired resistance) or death. To further corroborate our findings on limited numbers, immune gene expression profiling of all NSCLC samples from the TCGA database was also pursued.

## INTRODUCTION

Lung cancer is the leading cause of cancer-related mortality worldwide [1]. In recent years, treatment for non-small cell lung cancer (NSCLC) has dramatically evolved with the development of the anti-programmed death (PD-1) and anti-programmed death ligand 1 (PD-L1) checkpoint inhibitors. Historically, patients with metastatic disease have been treated with front-line platinum-based chemotherapy, with few efficacious options available at time of progression. Phase III studies have now demonstrated improved progression-free (PFS) and overall survival (OS) with PD-L1 inhibitors compared to second-line standard cytotoxic chemotherapy in both squamous and non-squamous NSCLC [2, 3]. In the front-line setting, for patients with at least 50% of tumor cells expressing PD-L1, the anti-PD-1 antibody pembrolizumab has demonstrated improvement in progression-free survival (PFS) and overall survival (OS) with fewer adverse events compared to standard chemotherapy [4]. Recently, a randomized phase II trial achieved an objective response when combining pembrolizumab and platinum-doublet chemotherapy compared to chemotherapy alone [5]. Further phase III studies of first-line therapy evaluating monotherapy, combination therapy, and immunotherapy combined with chemotherapy are ongoing.

Despite the success and advancements of checkpoint inhibitors, a majority of patients will experience innate or acquired (initial clinical benefit followed by the development of resistance) resistance. There remains a lack of information regarding predictive clinical and molecular markers of resistance. Most of the information available comes from other tumor types. At the molecular level, expression of PD-L1 in tumor cells and the presence of CD8+ infiltrating T cells within the tumor microenvironment have been associated with a trend towards clinical benefit [6, 7]. However, these remain as imperfect predictive biomarkers, likely due to the complexity of the relationship between the immune system and the tumor microenvironment. Composite gene expression profiling, demonstrating a more complete picture of the tumor biology, may represent a more optimal biomarker. Data in other tumor types suggests that a “T cell-inflamed” tumor microenvironment identified by immune gene expression studies correlates with an initial response to immunotherapy [8]. This microenvironment is characterized by infiltration of CD8+ T cells, chemokines, and other innate immune genes and has been found to have prognostic significance for response to immunotherapy [9, 10].

Given that PD1/L1 are becoming more prevalent in the treatment of lung cancer, further studies are necessary to identify clinical and molecular predictors of both innate and acquired resistance. In this study, we analyze the clinical characteristics of a cohort of NSCLC patients treated with anti-PD-1/PD-L1 monotherapy. We hypothesized that clinical characteristics would be predictive of either innate resistance, acquired resistance, or long-term benefit. Given the limited archival tissue available in these patient due to the combination of clinical trial participation and/or multiple diagnostic tests, we then chose to analyze the NSCLC cohort of The Cancer Genome Atlas (TCGA) to identify NSCLC subtypes associated with a “T cell-inflamed” microenvironment to further elucidate possible predictive biomarkers.

## RESULTS

### Baseline patient characteristics

A total of 93 NSCLC patients at the three sites were treated with an anti-PD-1/PD-L1 agent and were included in this retrospective study. Surviving patients were followed for a median of 11.5 months. All of the patients were treated off of any investigative protocol, except for two patients treated with the PD-L1 antibody atezolizumab.

Patient baseline characteristics for the entire cohort are described in Table 1. Mean age was 69 years (range 48-87 years). All patients had stage IV disease, with 32 (34.4%) having M1a disease and 61 (65.6%) with M1b. A significant proportion of patients, 91.4%, had 3 sites of disease or less. The majority of patients during this study received nivolumab (95.7%). Similar to phase III trials involving anti-PD-1/PD-L1 therapy, 14.0% had treated brain metastases prior to initiating therapy.

**Table 1:** Baseline clinical and demographic characteristics

Characteristic	No (*n* = 93)	%
**Age, Mean (range)**	69.1 (48–87)	-
**Sex**		
Male	48	51.6%
Female	45	48.4%
**Smoking History**		
Yes	86	92.5%
No	7	7.5%
Pack-years, Mean (range)^a^	35.7	(0-100)
**Histology**		
Adenocarcinoma	63	67.7%
Squamous	28	30.1%
Non-small cell carcinoma	2	2.2%
**Stage**		
M1a	32	34.4%
M1b	61	65.6%
**KPS^b^**		
0	12	13.0%
1	54	58.7%
2	23	25.0%
3	3	3.3%
**Brain metastases (Treated)**	13	14.0%
**Number of sites with at least one lesion**		
1	26	28.0%
2	40	43.0%
3	19	20.4%
4	6	6.4%
5	2	2.2%
**Mutational status^c^**		
KRAS	20	33.3%
EGFR	6	10.0%
ALK	1	1.7%
WT	33	55.0%
**Number of prior therapies**		
3	14	15.0%
2	37	39.8%
1	41	44.1%
0	1	1.1%
**Immunotherapy Agent**		
Nivolumab	89	95.7%
Pembrolizumab	2	2.2%
atezolizumab	2	2.2%
**Best Prior Response^b^**		
CR	6	6.5%
PR	31	33.7%
SD	26	28.3%
PD	29	31.5%
**Albumin, Mean (range)^d^**	3.4 (2.0-4.4	

^a^6 missing; ^b^1 missing; ^c^33 missing; ^d^18 missing.

Of the 93 patients, *n* = 36 patients (38.7%) had innate resistance and *n* = 57 (61.3%) had an initial clinical benefit. The median progression-free and overall survival times for the entire cohort were 5.4 and 11.0 months.

### Initial resistance characteristics

Compared to patients with an initial benefit, those with innate resistance were more likely to be non-smokers (30/36, *p* = 0.013) and smoked fewer pack-years (0.002), had more involved sites (*p* = 0.011), more prior therapies (*p* = 0.001), and a lower mean albumin level (*p* = 0.014) (Table 2). The two groups did not differ significantly with respect to any of the other baseline characteristics, although there was a trend toward higher KPS scores (*p* = 0.086) in the resistant group. We additionally found no consistent effects of line of therapy, particular drug or drug class on initial clinical benefit for anti-PD1/L1 treatment. All patients with EGFR or ALK mutation had received prior TKI per standard of care before receiving anti-PD1/L1. Chemotherapy regimens varied but were predominately based on platinum chemotherapy. Relative to radiation, we also found no impact on progression-free or overall survival for radiation either prior to immunotherapy or radiation at any point in the patients treatment course.

**Table 2:** Comparison of baseline characteristics in patients with primary resistance vs. Initial benefit

Characteristic	Primary resistance (*n* = 36)	Initial benefit (*n* = 57)	*P*-value
Age, Mean + SE	68.4 ± 1.6	69.6 ± 1.2	0.53
**Sex**			
Male	17 (47.2%)	28 (49.1%)	1.0
Female	19 (52.8%)	29 (50.9%)	
**Smoking History**			
Yes	30 (83.3%)	56 (98.2%)	0.013
No	6 (16.7%)	1 (1.8%)	
**Pack years, Mean + SE^a^**	26.7 ± 3.9	41.8 ± 2.8	0.002
**Histology**			
Adenocarcinoma	27 (75.0%)	36 (63.2%)	0.40
Squamous	0 (0.0%)	2 (3.5%)	
Non-small cell carcinoma	9 (25.0%)	19 (33.3%)	
**Stage**			
M1a	9 (25.0%)	23 (40.4%)	0.18
M1b	27 (75.0%)	34 (59.6%)	
**KPS^b^**			
0	3 (8.3%)	9 (16.1%)	0.086
1	18 (50.0%)	36 (64.3%)	
2	14 (38.9%)	9 (16.1%)	
3	1 (2.8%)	2 (3.6%)	
**Brain metastases (Treated)**	5 (13.9%)	8 (14.0%)	1.0
**Number of sites with at least one lesion**			
1	5 (13.9%)	21 (36.8%)	0.011
2	16 (44.4%)	24 (42.1%)	
3	8 (22.2%)	11 (19.3%)	
4	5 (13.9%)	1 (1.8%)	
5	2 (5.6)	0 (0.0%)	
**Mutational status^c^**			
KRAS	0 (0.0%)	1 (2.8%)	0.59
EGFR	3 (12.5%)	3 (8.3%)	
ALK	6 (25.0%)	14 (38.9%)	
WT	15 (62.5%)	18 (50.0%)	
**Number of prior therapies**			
3	12 (33.3%)	2 (3.5%)	0.001
2	12 (33.3%	25 (43.9%)	
1	12 (33.3%)	29 (50.9%)	
0	0 (0.0%)	1 (1.8%)	
**Immunotherapy Agent**			
Nivolumab	0 (0.0%)	2 (3.5%)	0.77
Pembrolizumab	35 (97.2%)	54 (94.7%)	
atezolizumab	1 (2.8%)	1 (1.8%)	
**Best Prior Response^b^**			
CR	2 (5.6%)	4 (7.1%)	0.33
PR	11 (30.6%)	20 (35.7%)	
SD	14 (38.9%)	12 (21.4%)	
PD	9 (25.0%)	20 (35.7%)	
**Albumin, Mean (range)^d^**	3.2 ± 0.12	3.5 ± 0.07	0.014

^a^6 missing; ^b^1 missing; c33 missing; ^d^18 missing.

### Acquired resistance characteristics

**Figure 1:**
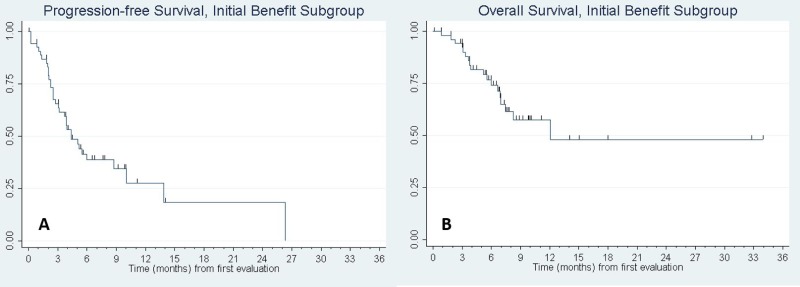
Kaplan–Meier curve of (**A**) progression-free survival and (**B**) overall survival of patients with initial benefit. *N* = 57 patients had initial benefit to anti-PD-1/PD-L1 monotherapy. Of these patients, median PFS was 4.4 months, 95% CI: (3.1, 8.7). Median OS was 12.1 months, 95% CI: (7.0, -). Tic marks denote censored observations.

**Figure 2:**
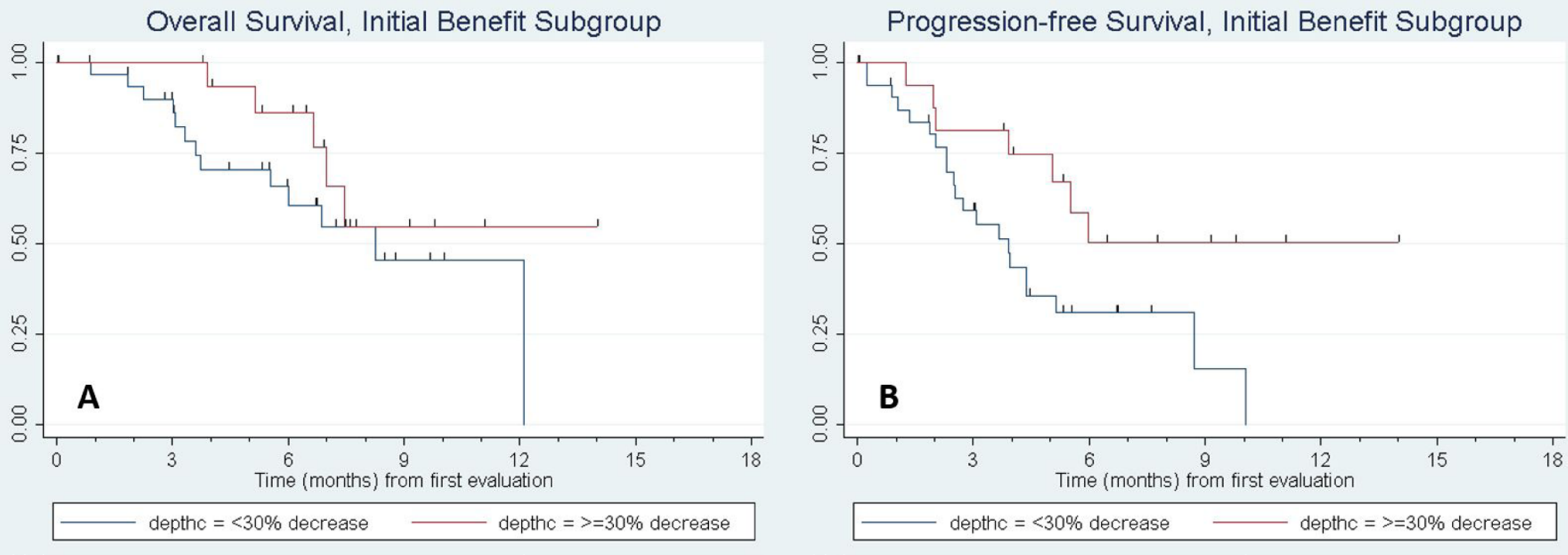
Kaplan–Meier curve of (**A**) progression-free survival and (**B**) overall survival of patients with initial benefit based on depth of response. The red curve denotes patients with ≥30% response in tumor size by RECIST criteria to anti-PD-1/PD-L1 therapy, while the blue line denotes response <30%. On multivariate cox regression analysis, PFS and OS were significantly longer for patients with increasing depth of response, with a hazard ratio (HR) of 0.74, 95% CI (0.62, 0.89), *p* = 0.002 for PFS and HR of 0.79 (0.65, 0.93), *p* = 0.006 for OS per every 10% increase in depth of response.

Of the fifty seven patients with initial clinical benefit, thirty four (59.6%) subsequently progressed (*n* = 33) or died absent a prior progression (*n* = 1). To assess factors associated with acquired resistance, we evaluated PFS and OS in the subgroup of patients who had initial clinical benefit. For this analysis, time was measured from the date of the initial CT evaluation and depth of tumor response to the anti-PD-1 agent was included among the predictor variables. Figure 1 shows the Kaplan–Meier curves. Supplementary Table 1, presents the results of fitting univariate Cox regression models for PFS and OS. Factors significantly associated with progression-free survival were KPS (*p* = 0.004) and depth of response to the anti-PD-1 therapy (*p* = 0.003). KPS was also significantly associated with overall survival (*p* = 0.010) and depth of response was marginally significant (*p* = 0.053). Molecular status specifically for KRAS mutation was tested and did not show a significant association with progression-free survival. Other mutations such as EGFR and ALK were not present in enough samples to support an analysis in these sub-populations. Multivariable analyses were then fit including univariate predictors significant at *p* < 0.15, followed by backward elimination until only statistical significant predictors remained. (Mutational status was omitted from the multivariable analyses due to a high rate of missing data (see Supplementary Table 2). KPS and depth of response remained in the final model for PFS; histology, number of involved sites, and depth of response were included in the final model for OS. PFS and OS curves for patients with a 30% or greater reduction in tumor size following anti-PD-1/PD-L1 treatment compared to those with less shrinkage are shown in Figure 2.

### Characteristics of progression and post-progression course

In patients with acquired resistance, the majority of patients (*n* = 20, 60.6%) had progression of existing disease rather than the development of new disease. Twenty two (66.7%) of the patients developed isolated (single organ) sites of progression, while *n* = 10 had more diffuse, systemic progression. All patients were receiving anti-PD-1 therapy at time of progression. Seventeen patients (51.5%) received further systemic therapy and 2 patients (6.1%) received local radiation therapy. The majority of these patients (*n* = 27, 81.8%) had progressive disease as best response to further systemic therapy. Four patients (12.1%) had a partial response. Local therapy was rare. The two patients that received radiation therapy have continued on active surveillance, and have not yet required systemic therapy after 9 months of surveillance.

### Immune gene expression profiling

**Figure 3:**
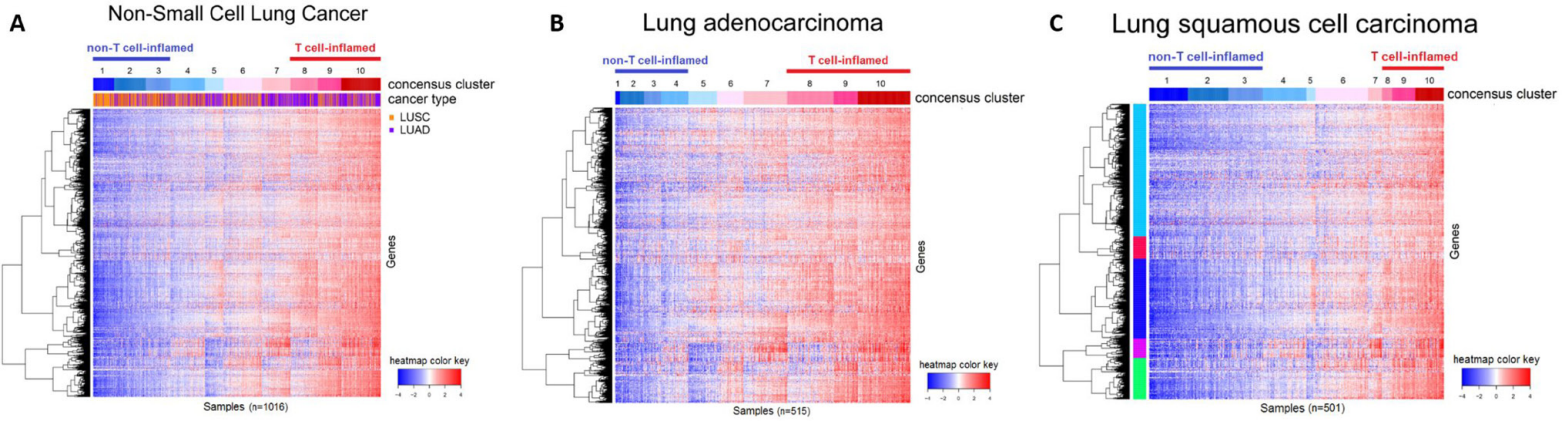
Immune gene expression heatmap of (**A**) all NSCLC samples, (**B**) lung adenocarcinoma (LUAC) samples, (**C**) lung squamous cell carcinoma (LUSC) samples. “T cell-inflamed” and “T cell-non-inflamed” tumors can be distinguished by immune gene expression profiling. In LUAC samples, 80 (15.53%) of the tumors showed minimal expression of T-cell-related immune genes (“T cell-non-inflamed”), whereas 215 (41.75%) show over-expression (“T cell-inflamed”). In LUSC samples, 193 (38.52%) showed minimal expression (“T cell-non-inflamed”) and 105 (20.96%) showed high expression (“T cell-inflamed”).

Through immune expression profiling of 1016 TCGA samples, three distinct tumor subtypes with low, moderate, and high expression level of the T-cell genes emerged. (Figure 3). Overall, the presence of the “T cell-inflamed” microenvironment was identified at a higher percentage in adenocarcinoma (42%) than squamous cell carcinoma patients (21%).

**Figure 4:**
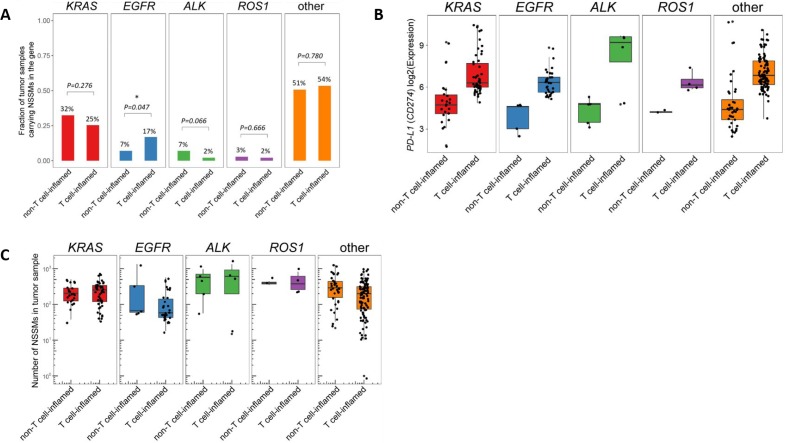
Analysis of lung adenocarcinoma samples based on molecular driver mutation (ALK, EGFR, KRAS, ROS1, or wild-type (none)) (**A**) “T cell-inflamed” versus “T cell-non-inflamed” samples, (**B**) PD-L1 (CD-47) expression, and (**C**) number of neoantigens were all evaluated. No significant differences in “T cell-inflamed” versus “T cell-non-inflamed” was observed based on molecular driver mutation, though a higher concentration of “T cell-inflamed” samples were detected in the EGFR samples that bordered on significance (17% versus 7%, *p* = 0.047). Similar level of PD-L1 expression and neoantigens were present regardless of mutation. Only mutually exclusive samples were included, samples with mutations in more than one driver mutation (*n* = 15) were not included. ^*^NSSMs = nonsynonymous somatic mutations (missense, nonsense, splice, insertions/deletions). Number on top of each bar represents the percentage of tumor samples carrying NSSM in each of the driver genes within non-T cell-inflamed or T cell-inflamed tumor group, which corresponds to the y-axis but in percentage units.

The proportion of adenocarcinoma tumor samples were further categorized by mutational subtype (KRAS, EGFR, ALK, or ROS1) and the proportion of “T cell-inflamed” versus “non-inflamed” was analyzed, as well as expression level of non-synonymous somatic mutations (NSSMs)—an indicator of mutational load—and PD-L1 expression (Figure 4). EGFR mutated sample demonstrated a higher proportion of “T cell-inflamed” tumors versus “non-inflamed” at marginal significance (17% versus 7%, respectively, *p* = 0.047). Otherwise, no significant differences were noted based on mutational subtype. There was no significant difference in expression of PD-L1 based on driver mutation, though ALK and ROS1 were not analyzed as they had too few samples. While on average, the mutational load was slightly lower in “inflamed” tumors relative to “non-inflamed” tumors in adenocarcinoma samples, further analysis of neoantigen load demonstrated no difference between “inflamed” versus “non-inflamed” subgroups in any of the driver mutation samples (see Supplementary Figure 1). Furthermore, data in melanoma has demonstrated no correlation between immune gene expression and either mutational load or neoantigen load [11].

## DISCUSSION

In this analysis of clinical and molecular factors influencing resistance to anti-PD1/L1 therapy in NSCLC we observed no clinical differences in innate resistance however noted multiple clinical factors associated with acquired resistance and additionally noted no differences in neoantigen load between T cell-inflamed and non-T cell-inflamed NSCLC tumors. In patients who developed acquired resistance, the median time to subsequent disease progression or death was 4.4 months beyond the time of their first CT evaluation. Factors associated with improved PFS in these patients were lower KPS score and depth of response to the anti-PD-1 therapy >30%. Factors associated with subsequent survival were adenocarcinoma histology, number of involved sites, and depth of response >30%.

Relevant to clinical practice, resistance was most often characterized by progression of an existing (rather than new) lesions. This progression was often isolated to a single site, rather than diffuse progression. Despite this confined progression, at our institutions local therapy appeared to be underutilized, with only 2 patient having radiation therapy after developing resistance. Pseudoprogression is a phenomenon first described in melanoma clinical trials suggesting that up to 15% of patients may manifest RECIST progressive disease prior to showing clinical benefit [12]. In NSCLC this phenomenon is much more rarely observed [13]. Clinical trials integrating multi-modal management of isolated sites of recurrence in NSCLC may be indicated to expand the benefit of PD1/L1 based immunotherapy.

From our cohort of NSCLC patients, little or no archival tissue was available due to prior clinical trial participation or standard diagnostic testing and we therefore turned to TCGA for interrogation of NSCLC at the molecular level. As TCGA captures patients prior to systemic therapy, we hypothesized that this sample set may be consistent on a molecular level with our analysis of pre-treatment factors associated with treatment resistance. In the NSCLC cohort of the TCGA database, a “T cell-inflamed” tumor microenvironment was more common in lung adenocarcinomas (42%), versus lung squamous cell carcinomas (21%). This will need to be investigated further in clinical trials, as current clinical data does not support a significantly more robust response in lung adenocarcinoma histologies. The TCGA analysis suggested that no molecular driver mutations are significantly associated with a “T cell-inflamed” tumor microenvironment, though data on EGFR mutated samples suggested a marginally significant increase in “T cell-inflamed” tumors (7% in “non-inflamed” versus 17% in “inflamed”). While similar signatures are highly associated with response to anti-PD1/L1 Ab, they are not perfectly predictive [7, 14]. The presence of the T cell-inflamed microenvironment in these EGFR mutant samples suggests that pure T cell-exclusion is unlikely to be a major mechanism of resistance. Alternative hypotheses could be resistance driven by increased presence of interferon-γ associated molecules such as secondary immune checkpoints including TIM3 [15] and/or Treg cells or alternatively activation of other immunosuppressive pathways such as CD73 [16, 17], among other possibilities.

The significant proportion of patients with initial clinical benefit in this study (61.3%) is significantly higher than the 19–20% response rates seen in large phase III trials investigating checkpoint inhibitors. While the reason for this is unclear, it is observed that patients in this retrospective study appeared to have less volume disease when initiating therapy (suggested by 91% of our patients having 3 sites of disease or less) compared to the subjects in these phase III trials.

This study describes key clinical criteria that may be predictive of innate resistance, acquired resistance, or death with anti-PD-1/PD-L1 therapy. Further investigations to confirm this data and better identify these patients is critical to help with treatment algorithms for this aggressive disease. The molecular analysis of the TCGA database suggests that lung adenocarcinoma tumors may be more commonly associated with a “T cell-inflamed” microenvironment, and thus possibly more likely to respond to immunotherapy. However, as of yet this has not been noted in larger, phase III clinical trials.

## MATERIALS AND METHODS

### Patients

Following approval from each site's institutional review board (IRB), we screened all patients diagnosed with stage IV NSCLC who received single-agent anti-PD-1/PD-L-1 therapy (nivolumab, pembrolizumab, or atezolizumab). The sites included the University of Chicago Cancer Center (*n* = 37), NorthShore University HealthSystems (*n* = 50), and Ingalls HealthSystems (*n* = 6). As the data were retrospective, waiver of consent was obtained at all sites. We included patients with both squamous and non-squamous histology who had received at least one dose of anti-PD-1 therapy. The schedule for radiographic tumor assessment for each patient was physician's choice, though typically CT scans were performed every 8 weeks unless clinically indicated. The start date of anti-PD-1/PD-L1 therapy for the first patient was 4/6/15 and data were last reviewed on 12/15/16.

### Study design

We obtained baseline demographic data for each patient including age, gender, American Joint Committee on Cancer (AJCC) pathologic stage, and performance status defined by the Eastern Cooperative Oncology Group (ECOG). Additional information regarding prior treatments and responses were also recorded. To assess the efficacy of initial anti-PD-1 therapy, we evaluated the objective response based on RECIST v1.1 criteria performed by the investigator, progression free survival (PFS), and overall survival (OS). Finally, to characterize the post-progression data, we collected data following disease progression including sites of progression, subsequent treatments, responses, and survival. Innate resistance was defined as disease progression by RECIST criteria on first CT evaluation or death prior to first CT evaluation; initial benefit was defined as alive with stable disease, partial, or complete response by RECIST criteria at first CT scan. Stable disease was included with response based on the hypothesis that the tumor microenvironment in patients with initial stable disease would be more similar to patients with initial response than those patients demonstrating initial progression. While this is an area of active translational research multiple clinical trials are on-going using this model [18, 19].

### Statistical analysis

Descriptive statistics (means, standard deviations, and percentages) were generated for baseline clinical and demographic data. OS and PFS were calculated based on the Kaplan–Meier method [20]. PFS was defined as time from the start of treatment until disease progression or death from any cause. Overall survival was defined as time from the start of treatment until death for any reason. Patients were censored at their last follow-up if no event had occurred. Ninety-five percent confidence intervals for the medians were derived using the method of Brookmeyer and Crowley [21]. Subsequent analyses were performed in two parts. First, we compared the baseline characteristics between patients with innate resistance, defined as disease progression on first CT evaluation or death prior to first CT evaluation, and patients with initial benefit, i.e., alive with stable disease, partial, or complete response at first CT scan. These comparisons consisted of two-sample *t*-tests for continuous variables and chi-square or Fisher's exact test for discrete data. Second, among the subset of patients with initial benefit, we constructed Kaplan–Meier curves and fitted univariate and multivariable Cox regression models [22] for PFS and OS to identify factors predictive of time to disease progression or death. Here, PFS and OS were measured from the time of the first CT scan, and candidate predictor variables included the initial depth of response to the treatment (percentage change in tumor size from baseline to first CT evaluation calculated on a continuous scale). In patients with initial benefit who subsequently progressed, their post-progression survival times were summarized using the Kaplan–Meier estimator.

### Analysis of TCGA data set

The Cancer Genome Atlas NSCLC level 4 RNA-seq gene expression data and level 2 somatic mutation data were downloaded from Broad Genome Data Analysis Center (http://gdac.broadinstitute.org) (release date 01/28/2016). A total of 515 adenocarcinoma (LUAD) and 501 squamous cell carcinoma (LUSC) primary tumors were analyzed. RNA-seq raw read counts were summarized at the gene level using the RSEM (RNA-Seq by Expectation Maximization) method, and were upper quartile-normalized and log_2_-transformed across all 1,016 samples in this study.

T-cell-inflamed and non-T-cell-inflamed group's tumor groups were identified by our previously described method [23]. In brief, unsupervised hierarchical clustering with K equal to 10 was performed on 17,867 genes that are expressed in at least 50% of the samples. A cluster of 943 genes including 12 genes of a previously described T-cell gene signature (CD8A, CCL3, CCL4, CXCL9, CXCL10, ICOS, IRF1, GZMK, HLA-DMA, HLA-DMB, HLA-DOA, and HLA-DOB) were selected for the identification of T cell-inflamed, intermediate, and non-T cell-inflamed tumor groups using a consensus clustering algorithm with 2,000 resampling cycles, unsupervised hierarchical clustering method and Euclidean distance [24] (ConsensusClusterPlus, v1.38.0). Whole exome somatic variants were filtered to retrieve NSSMs (non-synonymous somatic mutations) defined as missense mutations, nonsense mutations, small insertions/deletions, and those affecting splicing site of a protein-coding transcript. Predicted neoantigen data were obtained from a previous published study [25].

## SUPPLEMENTARY FIGURE AND TABLES


