# Supplementary material for: Clinical and molecular features of innate and acquired resistance to anti-PD-1/PD-L1 therapy in lung cancer
Source: Oncotarget. 2017 Dec 15;9(4):4375–84. doi: 10.18632/oncotarget.23315 (PMC5796980; doi:10.18632/oncotarget.23315)
Supplement: Supplementary file 1 [file oncotarget-09-4375-s001.pdf]

Clinical and molecular features of innate and acquired resistance to anti-PD-1/PD-L1 therapy in lung cancer

SUPPLEMENTARY MATERIALS

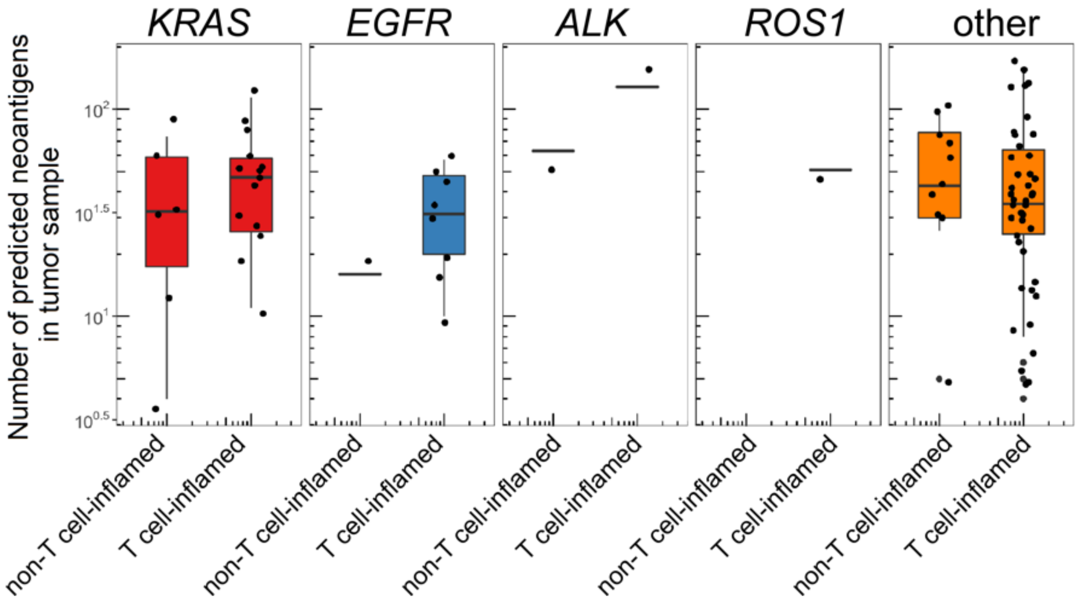

Supplmentary Figure 1: Analysis of neoantigen load on "inflamed" versus "non-inflamed".

Supplementary Table 1: Univariate cox regression analyses—Initial benefit subgroup (*n* = 57)

Covariate	PFS Hazard Ratio	95% CI	*P*-value	OS Hazard Ratio	95% CI	*P*-value
**Age (per 10 years)**	1.31	(0.87, 1.97)	0.20	1.60	(0.99, 1.11)	0.11
**Sex**						
Male	1.0 (ref)			1.0 (ref)		
Female	1.20	(0.60, 2.38)	0.60	1.23	(3.12, 0.48)	0.67
Pack-years (per 10 packyrs)a	1.01	(0.84, 1.23)	0.88	1.04	(0.82, 1.32)	0.75
**Histology**						
Adenocarcinoma	1.0 (ref)			1.0 (ref)		
Squamous/NSCLC	1.59	(0.78, 3.27)	0.20	2.20	(0.87, 5.59)	0.096
**Stage**						
M1a	1.0 (ref)			1.0 (ref)		
M1b	0.67	(0.34, 1.35)	0.26	0.92	(0.36, 2.36)	0.87
**KPSb**						
0/1	1.0 (ref)			1.0 (ref)		
2/3	3.41	(1.48, 7.85)	0.004	3.70	(1.36, 10.0)	0.010
**Brain metastases (treated)**						
No	1.0 (ref)			1.0 (ref)		
Yes	0.83	(0.25, 2.76)	0.76	1.95	(0.55, 6.82)	0.30
Number of involved sites	0.99	(0.62, 1.58)	0.97	1.74	(0.94, 3.25)	0.078
**Mutational statusc**						
WT	1.0 (ref)			1.0 (ref)		
KRAS, EGFR, or ALK	2.76	(1.13, 6.75)	0.026	2.02	(0.59, 6.90)	0.26
Number of prior therapies	1.06	(0.63, 1.80)	0.82	1.45	(0.71, 2.99)	0.31
**Best Prior Responseb**						
CR/PR	1.0 (ref)			1.0 (ref)		
SD	1.83	(0.78, 4.28)	0.16	1.29	(0.39, 4.33)	0.68
PD	0.88	(0.39, 2.00)	0.77	1.06	(0.36, 3.07)	0.92
Albumin (per unit)d	0.76	(0.37, 1.55)	0.45	0.76	(0.30, 1.92	0.56
Depth of response to anti-PD-1 (per 10% decrease)e	0.78	(0.67, 0.92)	0.003	0.84	(0.70, 1.00)	0.053

[a]5 missing; [b]1 missing; [c]21 missing; [d]6 missing; [e]7 missing.

Supplementary Table 2: Multivariate cox regression analyses—initial benefit subgroup (*n* = 57)

Covariate	PFS[a]			OS[a]		
	Hazard Ratio	95% CI	*P*-value	Hazard Ratio	95% CI	*P*-value
**Histology**						
Adenocarcinoma				1.0 (ref)		
Squamous/NSCLC				3.21	(1.17, 8.86)	0.024
**KPS[b]**						
0/1	1.0 (ref)					
2/3	4.30	(1.75, 10.6)	0.001			
**Number of involved sites**				2.23	(1.14, 4.37)	0.020
**Depth of response to anti-PD-1 (per 10% decrease)[c]**	0.74	(0.62, 0.89	0.002	0.78	(0.65, 0.93)	0.006

[a]7 missing.
